# A case report of two siblings with Alstrom syndrome without hearing loss associated with two new *ALMS1* variants

**DOI:** 10.1186/s12886-019-1259-y

**Published:** 2019-12-07

**Authors:** Maria F. Shurygina, Maria A. Parker, Catie L. Schlechter, Rui Chen, Yumei Li, Richard G. Weleber, Paul Yang, Mark E. Pennesi

**Affiliations:** 1 S. Fyodorov Eye Microsurgery Federal State Institution, 59A, Beskudnikovsky Blvd, Moscow, 127486 Russia; 2 Casey Eye Institute, Oregon Health & Science University, 3375 SW Terwilliger Blvd, Portland, OR 97239 USA; 3 Human Genome Sequencing Center, Department of Molecular and Human Genetics, Baylor College of Medicine, Houston, TX USA

**Keywords:** Alström syndrome, Cone-rod dystrophy, *ALMS1* gene, Case report

## Abstract

**Background:**

Alström syndrome (AS) is a rare monogenic disorder characterized by progressive multi-organ pathology including retinal degeneration, hearing impairment and type 2 diabetes. Here we present clinical features in two siblings diagnosed with Alström syndrome associated with two novel changes in *ALMS1*.

**Case presentation:**

Two siblings originally diagnosed as having achromatopsia presented with mild light sensitivity, nonspecific otitis media, and mild developmental delay during the first decade of life with a relatively stable ocular appearance during second decade, late onset of nystagmus and dyschromatopsia (after 20 years) and preserved vision during the third decade of life. One sibling had late onset hearing loss and both siblings had symmetric high myopia, normal stature, and ptosis. Clinical findings revealed structural and functional tests consistent with a cone-rod dystrophy. Novel variants c.9894dupC (p.S3298 fs) and c.10769delC (p.T3590 fs) in *ALMS1* gene were found.

**Conclusions:**

Two North American siblings who presented with a mild clinical phenotype of Alström syndrome were found to have novel mutations in *ALMS1*. These two frame-shift mutations segregated with the disease phenotype lending evidence to their pathogenicity.

## Background

Alström syndrome (AS) is a rare monogenic disorder characterized by progressive multi-organ pathology. The diagnosis is based on the presence of major and minor criteria. Major criteria include: a pathogenic mutation in one *ALMS1* allele/or family history of Alström syndrome, nystagmus, legal blindness, cone-rod dystrophy. Minor criteria include obesity and/or insulin resistance and/or type 2 diabetes mellitus, dilated cardiomyopathy with congestive heart failure, hearing loss, hepatic dysfunction, renal failure, short stature, hypogonadism (males), hyperandrogenism (females) and other variable supportive evidence [1]. AS often leads to organ failure, resulting in a reduced life expectancy, rarely exceeding 50 years [2]. Among non-consanguineous populations of America and Europe, the prevalence of AS is estimated to be approximately 1:1,000,000 [2]. However, the rate of AS may be increased in populations that are geographically isolated or have a high rate of consanguinity [3–5]. Male and female individuals are affected with equal probability (1:1 ratio) and there is no one ethnic group more likely to carry *ALMS1* mutations [1].

The clinical presentation of AS is heterogenous with different levels of severity and rates of progression. Ocular manifestations usually occur in the first decade of life and include: nystagmus, photosensitivity, and decreased visual acuity [1, 6]. Approximately one-third of patients are completely blind by age nine, 50% are blind by age 12 and 90% are blind by age 16 [1]. Greater than 85% of patients have sensorineural hearing loss, which usually presents in the first decade of life and is often compounded by chronic otitis media. By the age of 16 approximately 95% of patients have Type 2 diabetes and without dietary modifications almost 100% are obese [1, 2, 7].

The *ALMS1* gene is located on chromosome 2p13 [8]. To date 239 disease-causing mutations in *ALMS1* have been identified [3]. The majority (96%) of the mutations are nonsense and frameshift variations that are predicted to cause premature protein truncation. The function of the protein encoded by the *ALMS1* gene is not well understood, but it has been implicated to play a role in ciliary function, cell cycle regulation, microtubule organization, and intracellular transport. This supports the inclusion of AS in a group of genetic disorders known as ciliopathies [9–13]. Unlike, other ciliopathies, such as Bardet-Biedel syndrome, triallelic inheritance has not been reported [3]. Previous reports have shown ‘hot spots’ for deleterious mutations in exon 8, exon 10, and exon 16 [2, 14]. *ALMS1* is expressed in all tissues that are pathologically affected in patients with AS [9–12].

Here we present the clinical features in two siblings with symptoms consistent with AS and associated with two novel *ALMS1* variants.

## Case presentation

Patient 1 was a 29-year-old male. He was referred to the OHSU Casey Eye Institute for evaluation of poor vision, increased light sensitivity, nystagmus and difficulty seeing colors. He reported seeing much better in low light, denied nyctalopia and needed assistance when walking in daylight or bright light areas. He was born from full term pregnancy without complications. There was no history of consanguinity. The first ophthalmological assessment was at age 3, at which bilateral optic nerve pallor, myopia, astigmatism, low vision and light sensitivity were noted. Visual acuity had not changed significantly during first two decades of his life. He had a past medical history attention deficit disorder and delirium. A follow-up visit at age 12 showed abnormal responses of visual evoked potentials but a MRI scan was negative for any intracranial abnormalities. The patient first noted dyschromatopsia at age 27. He was diagnosed with balance problem and testosterone deficiency at age 21 and diabetes mellitus type 2 at age 29. He has had one episode of the otitis media during his childhood, but had normal hearing. At his most recent exam his height was 6 ft., weight was 225 lb. (less than 85th percentile), and the body mass index (BMI) was 30.5. He had no cardiac history, renal or hepatic dysfunctions or polydactyly.

**Fig. 1:**
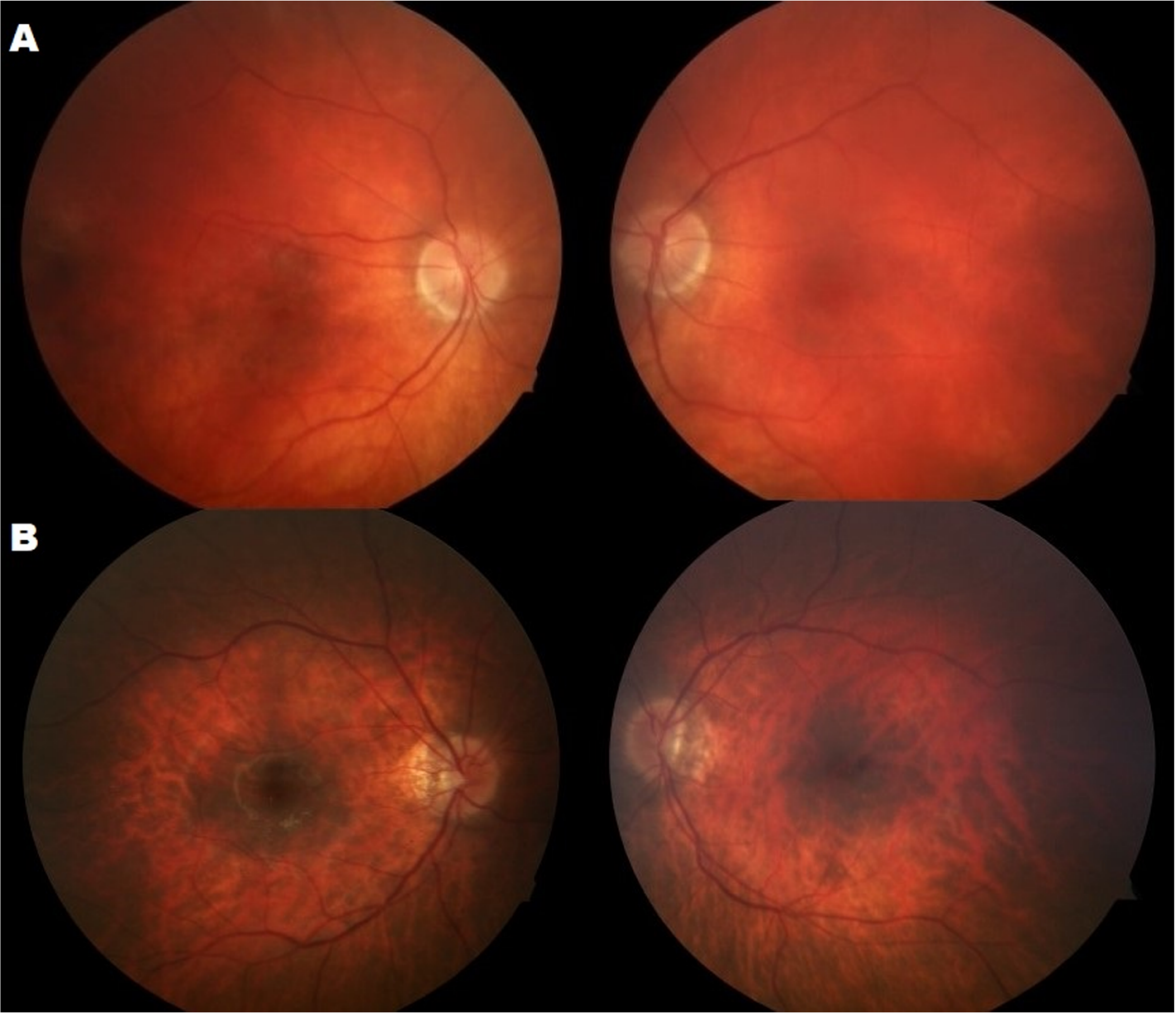
The color images showed fundus tessellation and subtle pigment mottling in both eyes (**a** – Patient 1, **b** – Patient 2)

**Fig. 2:**
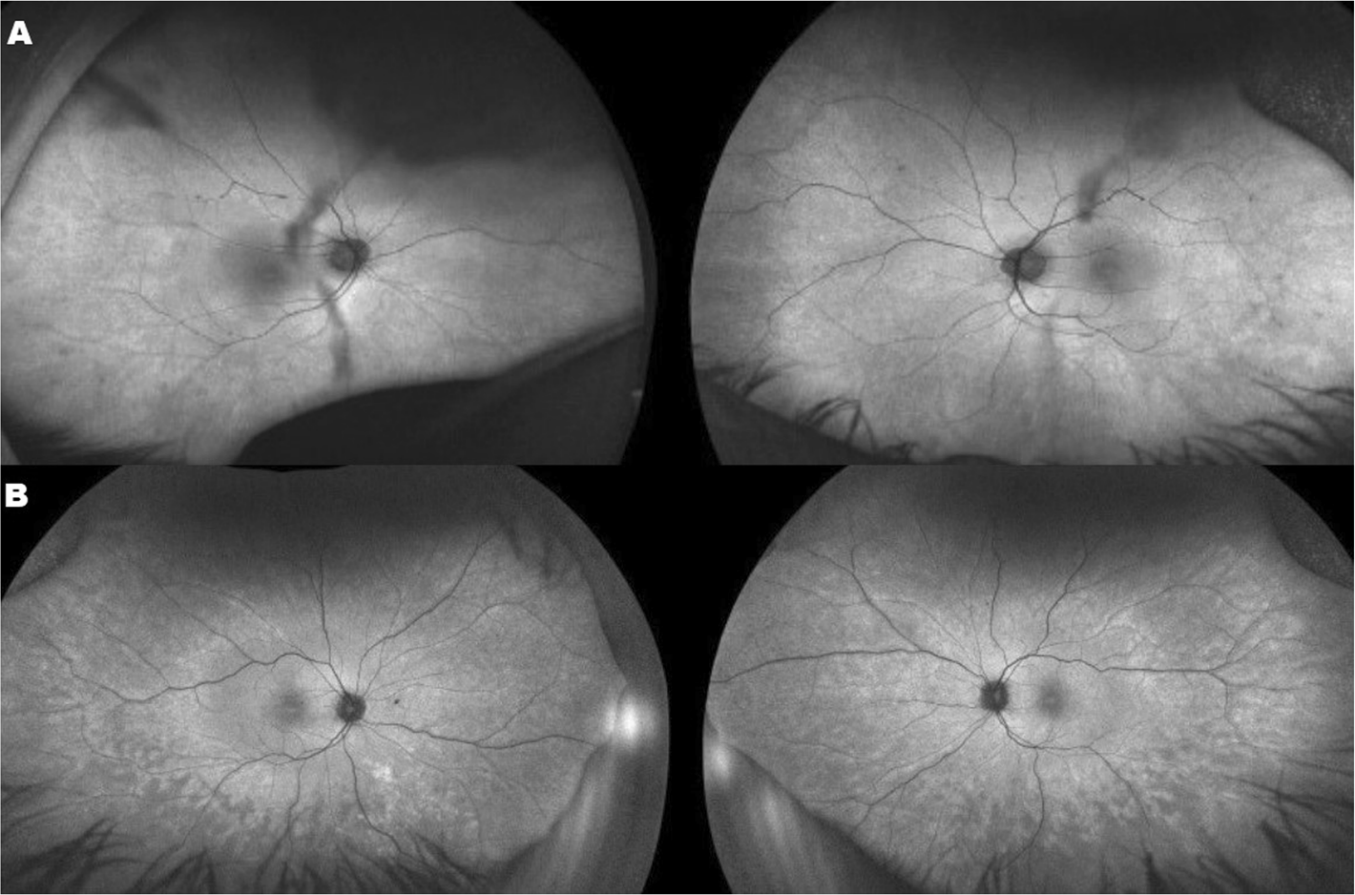
Fundus autofluorescence (FAF) images of both eyes showed (**a** – Patient 1) subtle irregular hyper-autofluorescence at the posterior pole and several hypo-autofluorescence spots on the retina because of the shadows from the posterior subcapsular cataract. (**b** – Patient 2) subtle irregular hyper-autofluorescence at the posterior pole with patches of relative hypo-autofluoresence in the periphery

**Fig. 3:**
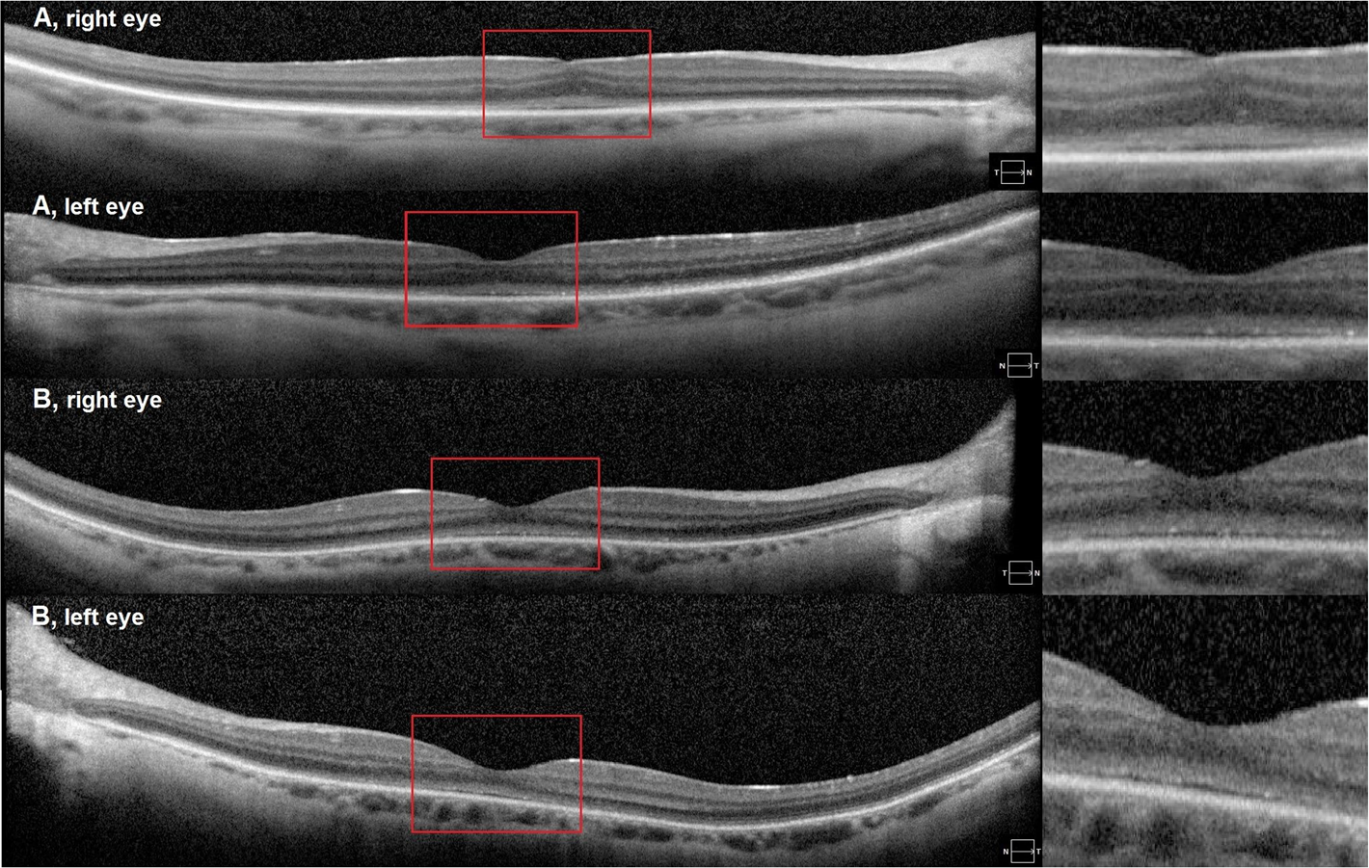
Spectral domain optical coherence tomographic (SD-OCT) images of both eyes in patient 1 (A) and patient 2 (B) showed: foveal hypoplasia, decreased reflectivity of the EZ-band, indistinct ELM and presence of hyporeflective area underneath the EZ-band foveal hypoplasia

Snellen visual acuity measured 20/200 OU and 20/150 OS. Manifest refraction revealed high myopia with astigmatism (− 8.75 + 3.50 × 95 right eye and − 8.00 + 3.50 × 80 left eye). The patient failed all plates from the HRR test demonstrating severely impaired color vision (dyschromatopsia)*.* Extraocular movements were full but there was a low amplitude nystagmus. Anterior segment exam reveal ptosis in the right eye, posterior subcapsular cataracts both eyes, and rare cells in the anterior vitreous, but there cannot rule out the vitreous opacities. Fundoscopy revealed normal appearing optic nerves, mild vascular attenuation and subtle macular pigment mottling (Fig. 1a). FAF demonstrated a subtle hyperautofluoresent spot in the fovea of both eyes and areas of hypoautofluoresent spots in the periphery (Fig. 2a). Horizontal cross-sectional SD-OCT line scans showed Grade 2 foveal hypoplasia right eye Grade 1 left eye (Fig. 3a) [15]. There was decreased reflectivity of the ellipsoid zone (EZ) in the fovea, an indistinct the external limiting membrane (ELM), presence of subfoveal hyporeflective zone, and thinning of the outer nuclear layer (ONL).

**Fig. 4:**
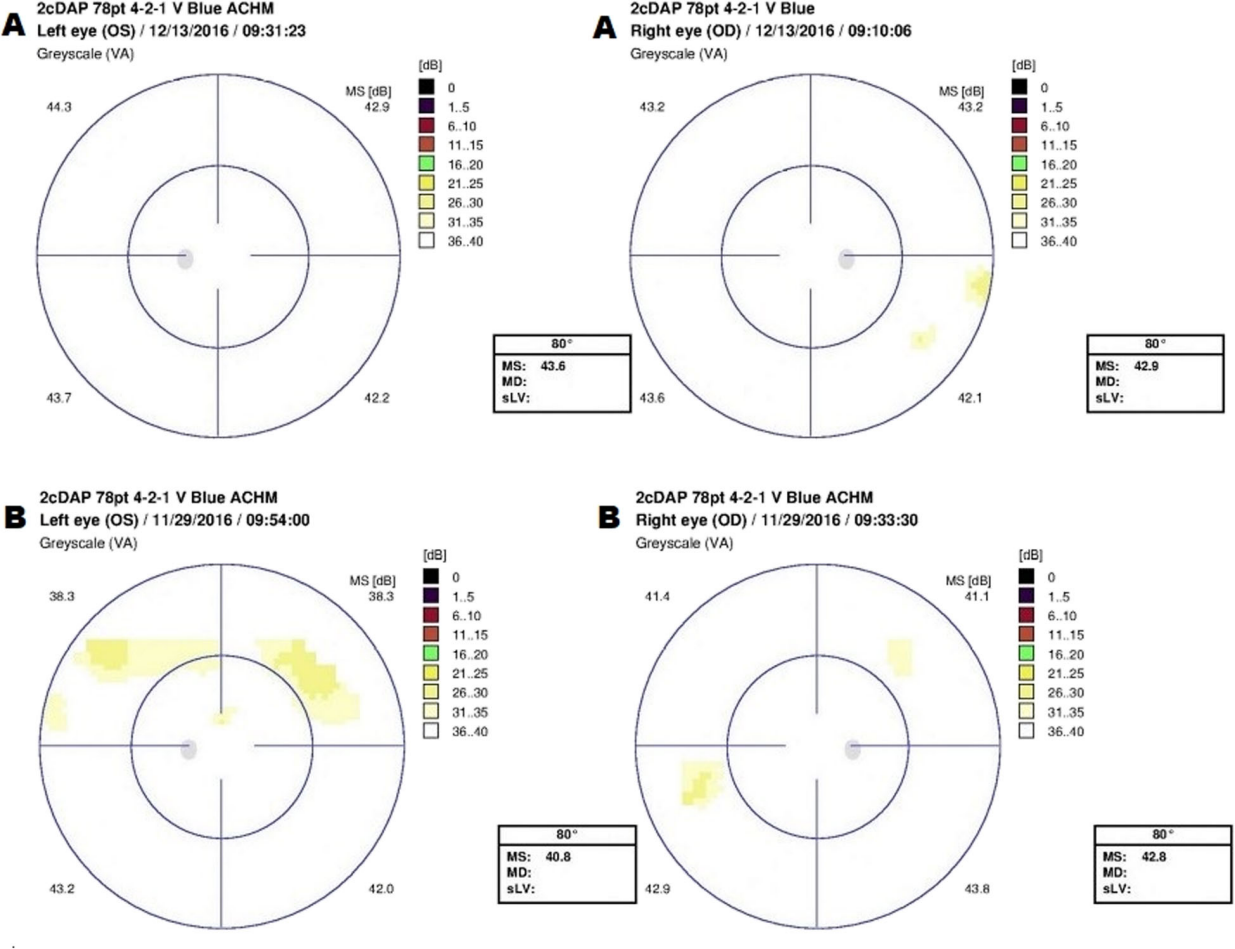
Static dark adapted perimetry with blue stimulus of both eyes for A – Patient 1 and B – Patient 2. Visual field shows good sensitivity of both eyes in patient 1 (A). Patient 2 (B) showed good sensitivity with only two small areas of relatively decreased sensitivity in both eyes

**Fig. 5:**
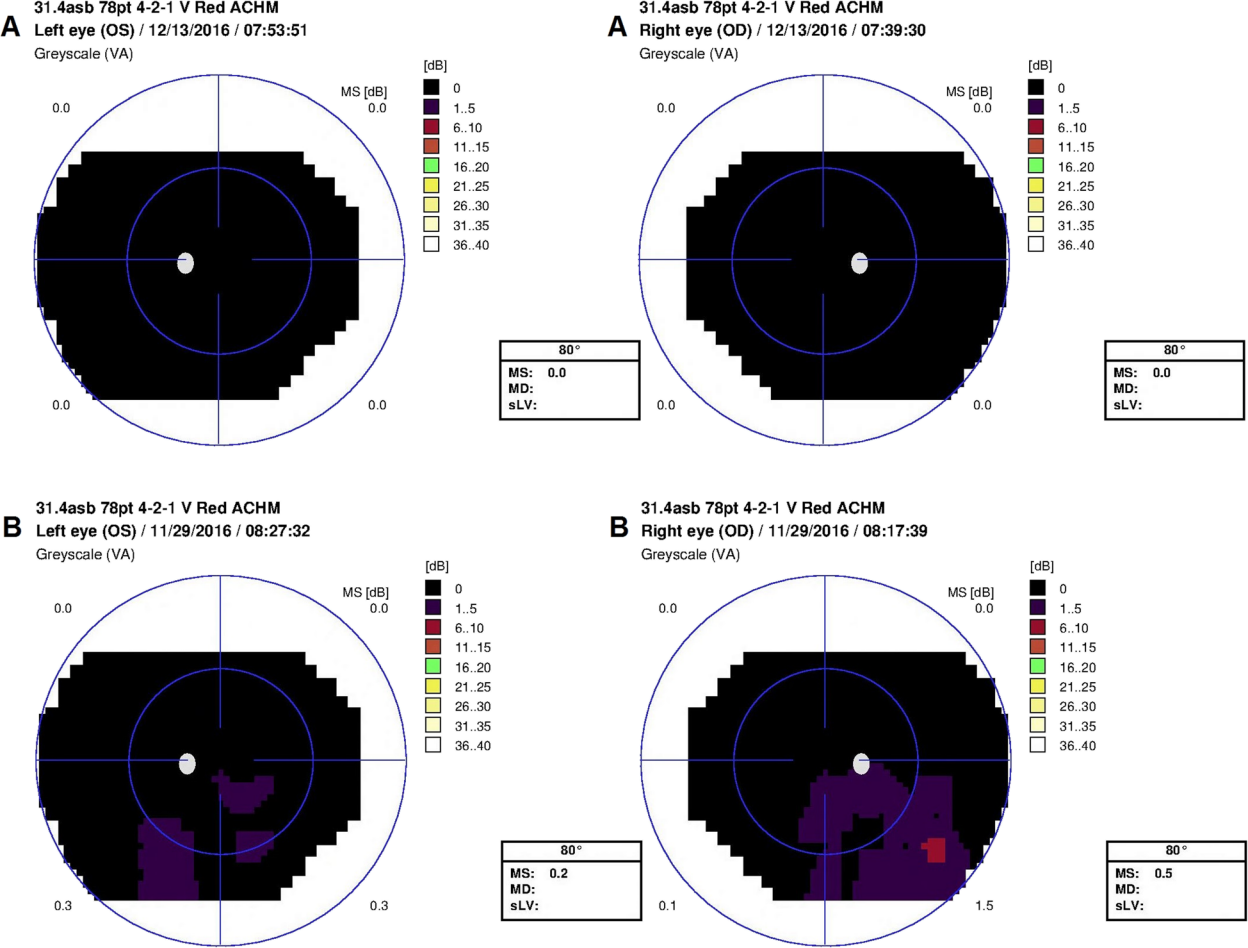
Static light adapted perimetry with red stimulus of both eyes for A – Patient 1 and B – Patient 2. Visual field shows severely attenuated cone sensitivity to a red stimuli under light adapted conditions (both patients). A: Sensitivity to the red stimulus was essentially undetectable in both eyes (Patient 1). B: Exam shows several small areas of residual sensitivity in inferior visual field in both eyes

Two-color perimetry demonstrated that rod sensitivity was relatively intact. The mean sensitivity measured by static perimetry with the blue stimuli under dark adapted conditions was 42.9 dB for the right eye and 43.6 dB for the left eye (Fig. 4a). In comparison, cone sensitivity was severely attenuated with essentially unrecordable responses in both eyes to a red stimuli under light-adapted conditions (Fig. 5a).

**Fig. 6:**
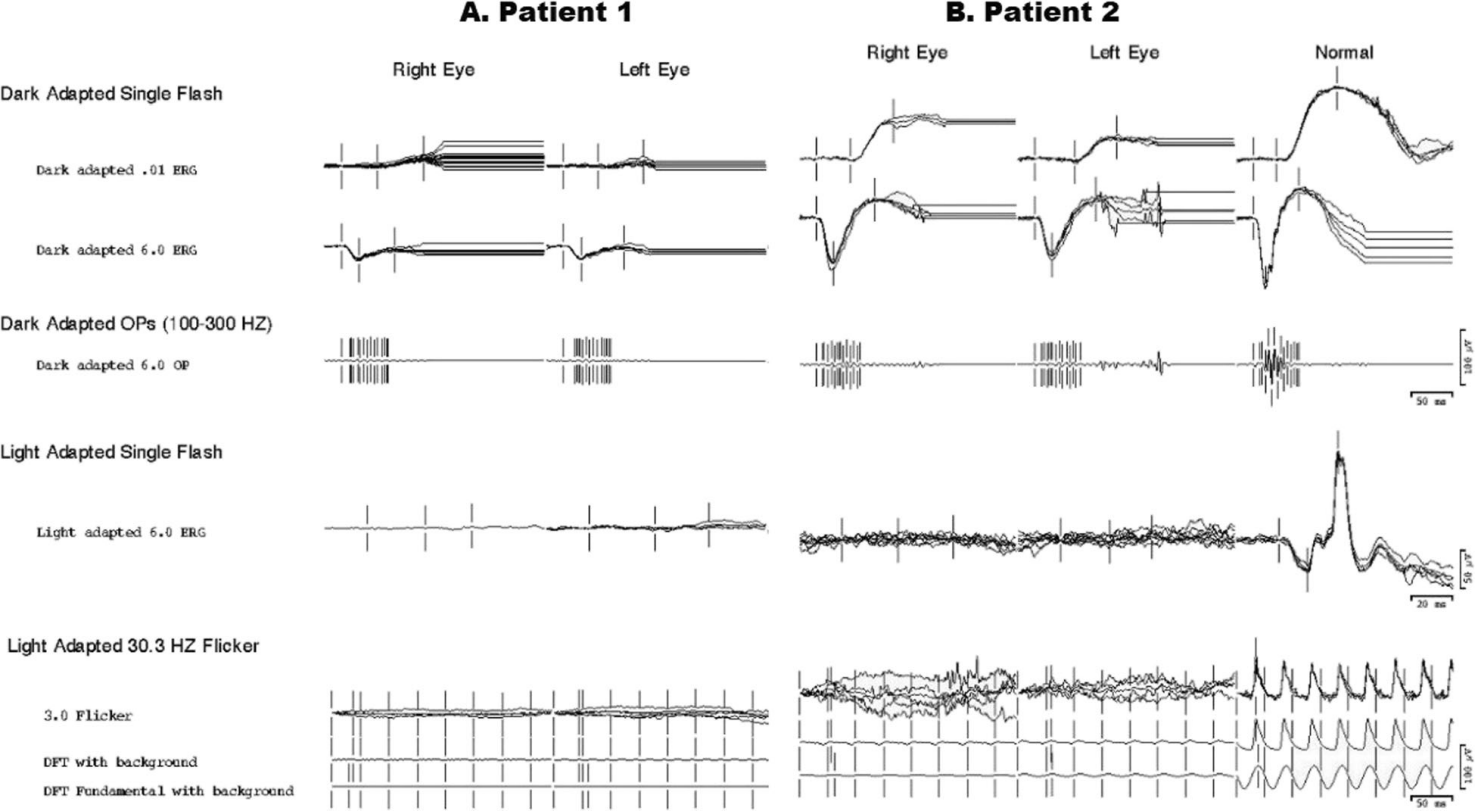
Full-field ERGs responses are shown for A – Patient 1 and B – Patient 2. The rod responses are moderately to severely decreased for both patients (A and B). Cone responses to LA 3.0 and 30 Hz flicker stimuli were severely attenuated in both patiernts

**Fig. 7:**
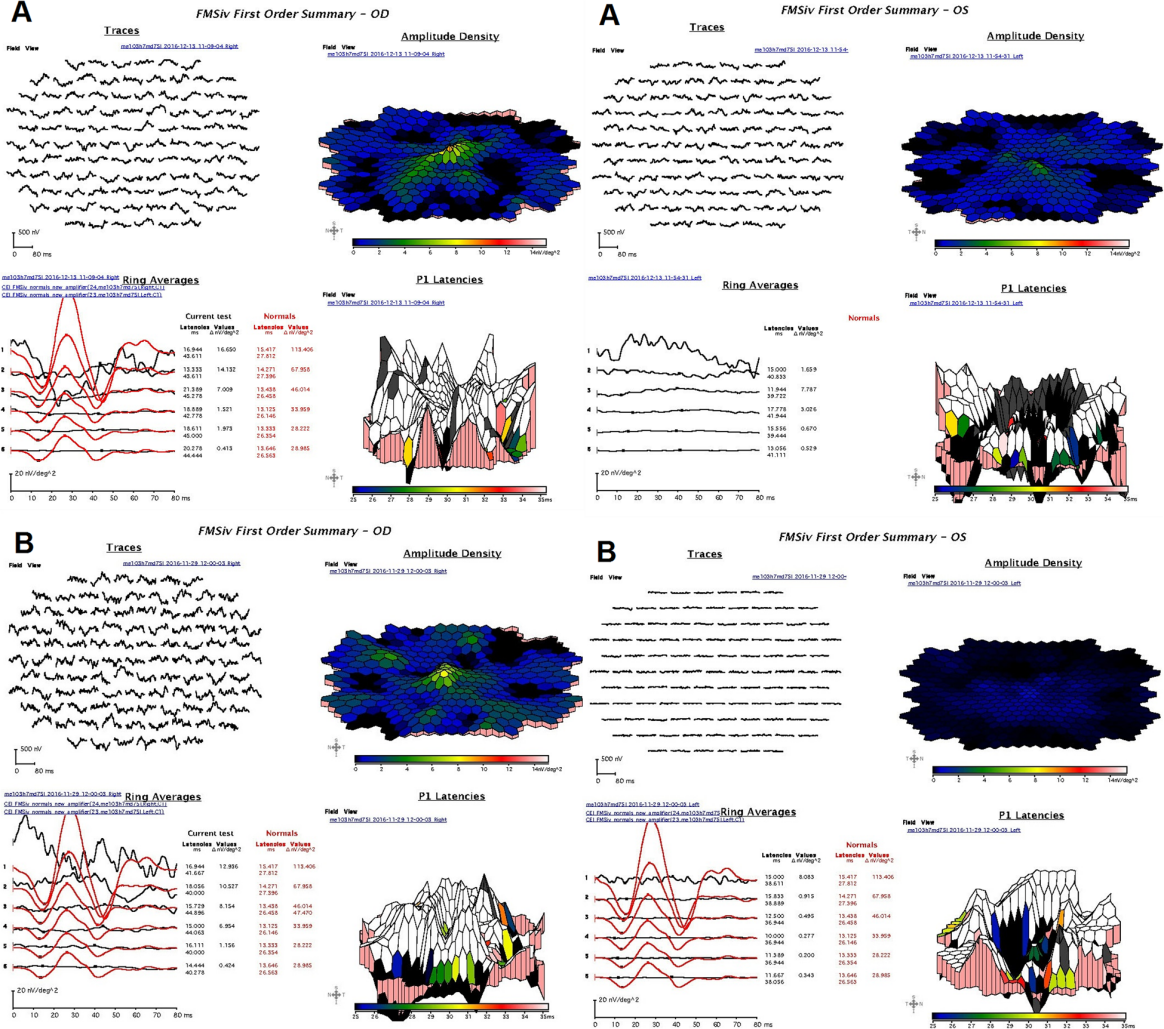
Multifocal ERGs of both eyes for **a** – Patient 1 and **b** – Patient 2. The local first-order response P1amplitude arrays (scalar-product) shows severely diminished amplitude densities in both eyes (A and B)

Full-field electroretinogram showed moderately to severely decreased rod responses, unrecordable photopic responses in both eyes and prolonged timing in all responses (Fig. 6a). The photopic light adapted 30 Hz flicker ERGs showed unrecordable responses both eyes. There were small signals possibly coming from dark adapted cones, but this could also be rod contamination. These ERG results indicated an absence of cone function and severely decreased rod function even once the effect of high myopia is taken into consideration [16, 17]. The local first-order response P1-amplitude response arrays (scalar-product) from the multifocal ERG showed unrecordable responses in both eyes (Fig. 7a) consistent with severely decreased macular cone function.

Patient 2, a 24-year-old male, is the sibling of patient 1 who was referred for evaluation of poor vision, dyschromatopsia, and photosensitivity starting at the age of two. He was born from full term pregnancy without complications. The first ophthalmology assessment at age two noted bilateral optic nerve pallor, myopia, astigmatism, low vision, mild light sensitivity (photoaversion) and strabismus. Visual acuity had not changed significantly during first two decades of his life. He had history of developental delay. Dyschromatopsia was first noted at age 22. Since early childhood he was diagnosed with chronic otitis media and with mild hearing loss at age 23. His height was 6 ft., weight was 242 lb. (less then 90th percentile), and his BMI was 32.8. He was diagnosed with balance problems, asthma, growth retardation and also has history of seizures. He has no cardiac history, diabetic, renal or hepatic dysfunctions and polydactyly.

Snellen visual acuity measured 20/150 in both eyes. Manifest refraction revealed high myopia with astigmatism (− 12.50 + 3.50 × 90 right eye and − 12.50 + 3.25 × 75 left eye). The patient failed all plates from the HRR test demonstrating severely impaired color vision*.* Extraocular movements were full without nystagmus. Anterior segment exam reveal ptosis in both eyes, posterior subcapsular cataracts both eyes, and rare cells in the anterior vitreous. Fundoscopy revealed tilted and small appearing optic nerves, mild vascular attenuation and macular pigment mottling and peripheral retinal atrophy (Fig. 1b). FAF demonstrated a subtle hyperautofluoresent spot in the fovea of both eyes and areas of hyper/hypoautofluoresent patches in the periphery (Fig. 2b). Similarly to his brother (patient 1) OCT showed: foveal hypoplasia, decreased reflectivity of the EZ-band, indistinct ELM and presence of hyporeflective area underneath the EZ-band (Fig. 3b).

Mean sensitivity measured by static perimetry with the blue stimuli were 42.8 dB and 40.8 dB for right and left eye respectively (Fig. 4b); and with the red stimuli 0.5 and 0.2 dB (right and left respectively) (Fig. 5b).

Full-field ERGs showed moderately decreased rod responses (Fig. 6b). The photopic ERGs, light adapted 3.0 and 30 Hz flicker showed severe, almost complete attenuation of cone photoreceptors amplitude in both eyes (Fig. 6b) with prolonged timing in all responses. These ERG results indicated an absence of cone function and severely decreased rod function even after high myopia is taken into consideration. The local first-order response P1-amplitude response arrays (scalar-product) of multifocal ERG showed unrecordable densities for both eyes, more significant for left eye (Fig. 7b).

**Fig. 8:**
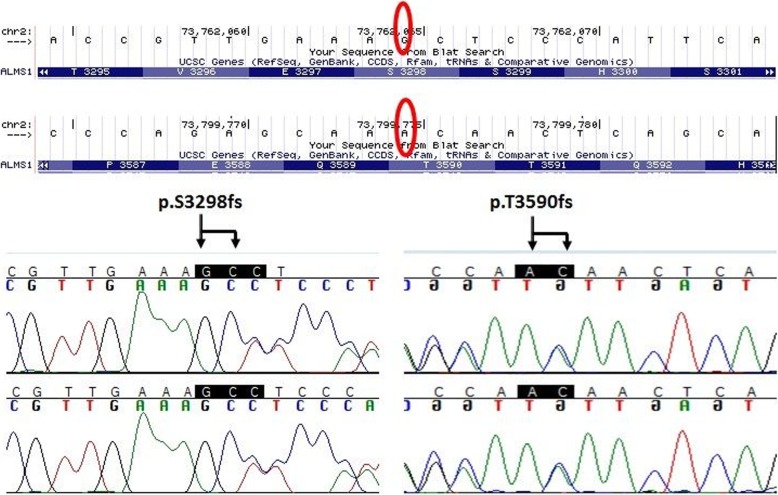
Two different mutations in *ALMS1* were identified in patient 1 and his affected brother patient 2, c.9894dupC:p.S3298 fs and c.10769delC:p.T3590 fs

Targeted-Capture Retinal Panel analysis identified 8 rare variants in patient 2, including *HMCN1* (c.G2119 T:p.V707F)*, TTC21B (c.A234G:p.I78M) KIAA1549 (c.G4781A:p.R1594Q), BBS1 (c.A53G:p.E18G), CEP290 (c.A2568G:p.I856M), ALMS1* (c.41_42insGGA:p.E14delinsEE; c.9894dupC:p.S3298 fs; c.10769delC:p.T3590 fs). Based on the nature of the variants, gene function, and HGMD and Clinvar database search, the two novel frameshift mutations in *ALMS1* are most likely the disease causing mutant alleles in the patient. Interestingly, the same *ALMS1* mutations, one insertion c.9894dupC (p.S3298 fs) and one deletion variant, c.10769delC” (p.T3590 fs) (Fig. 8) were also identified in patient 1. Due to the distance between two variant about 40Kb apart, there not feasible to run long range PCR. The segregation test was not performed due to the father’s refusal to provide a blood sample.

## Discussion and conclusions

Alström syndrome most commonly presents with a cone-rod dystrophy, progressive sensorineural hearing loss, short stature, obesity, cardiomyopathy and various degrees of metabolic disturbances [6, 18–21]. Most symptoms are typically begin in the first decade of life but there is a severety of the phenotypic spectrum [22].

Here we describe two siblings who presented with decreased central vision, dyschromatopsia, photophobia, high myopia, and cone-rod dysfunction. Our patients demonstrated mild phenotypes of many of the typical clinical symptoms of AS leading to a delayed diagnosis. For both cases, the initial symptom was mild light sensitivity with onset at two years of age. Based on the fundus appearance and visual ability, the patients were originally given a diagnosis of myopia and optic atrophy. During first decade of the life, the patients had a relatively stable ocular appearance. Based on the history of early onset light sensitivity, high myopia, nystagmus, decreased visual acuity, decreased of color vision, and cone dysfunction much greater than rod dysfunction on ERG, our initial differential diagnosis was achromatopsia versus a cone-rod dystrophy. However, research genetic testing revealed two novel frameshift variants in *ALMS1* in both brothers. The first was a single base pair duplication c.9894dupC (p.S3298 fs, NM_015120, exon 12) and the second a single base pair deletion c.10769delC (p.T3590 fs, NM_015120, exon 16). Upon further systemic review, additional findings consistent with Alstrom syndrome were found including: history of otitis media (Patient 1 and 2), mild developmental delay (Patient 1 and 2), type 2 diabetes mellitus (Patient 1), testosterone deficiency (Patient 1) and mild hearing loss (Patient 2).

Most pathogenic variants in *ALMS1* occur downstream from exon 7, with the ‘hot spots’ for disease-causing variants in exons 8, 10, and 16 [2, 3, 14]. Both our variants were not previously described, but, there are two pathogenic mutations located close to these variants [14, 23]. One of these variants, c.10775delC (exon 16), was the most frequently identified in patients of English descent, suggesting a possible founder effect [14]. In addition, we know that majority of the mutations that are nonsense and frameshift variations are predicted to cause premature protein truncation, resulting in the early termination of *ALMS1* or a non-functional protein [3].

Our diagnostic findings: visual fields, ffERG, mfERG, SD-OCT, fundus appearance are consistent with typically clinical findings in a cone-rod dystrophy as usually seen in AS.

There are several interesting clinical findings our patients. Consistent with a mild systemic phenotype,neither of our patients had cardiac, renal or hepatic dysfunction. Nearly 89% of patients with AS have hearing loss with average age of detection of 7.45 years (range 1.5–15), predominantly symmetric, sensorineural, that may progress to a severe degree [24]. Only one of our patients had a history of chronic otitis media with mild hearing loss that did not manifest until the third decade. Nearly 82% patients with AS have type 2 diabetes mellitus with the median age of onset 16 years, but only one sibling had diabetes mellitus type 2 that was not diagnosed until age 29 [1, 3, 7]. Unlike most patients with AS, where the height is normal in early childhood, but stalls in adolescence, our patients have normal stature (50-75th centile) [3]. Obesity generally begins to develop in the first few years of life and without weight control the BMI of most young children is >95th percentile (3). Our patients have weight less than the 90th percentile. Additionally, our patients both presented with high myopia, but previous studies primarily reported hyperopia in AS, ranging from mild to high, and mentioned only two cases of myopia [6, 18–21, 25–28]. Our patients have no family history of myopia, indicating that its development may be specific to these novel variants in *ALMS1*. Finally, our patients have also had the presence of ptosis in both eyes and this feature not described before AS patients.

Currently, there is no known cure for AS other than managing underlying systemic diseases. The supportive treatments are available to manage symptoms and include a diet high in anti-oxidants, correction of refractive error, low-vision aids and wearing sunglasses outside. Although we only report two cases of the rare condition we have provided detailed clinical features and genotype-phenotype correlation in the two novel frameshift variants in *ALMS1* that were never described before to our knowledge*.* The two new variants result in a mild phenotype, delayed onset, presence of high myopia, ptosis, late onset of hearing loss, and mild systemic features. We recommend a thorough systemic evaluation coupled with diagnostic and genetic testing in patients with achromatopsia/cone-rod dystrophy to avoid misdiagnosis.

## Data Availability

The datasets used and/or analysed during the current study are available from the corresponding author on reasonable request.

## Abbreviations

AS: Alström syndrome
FAF: Fundus autofluorescence
ffERG: Full-field electroretinography
mfERG: Multifocal electroretinography
SD-OCT: Spectral domain optical coherence tomography
